# 

**Published:** 1843-10

**Authors:** 


### CONTENTS OF No. XXXII.

OF THE

### British and Foreign Medical Review

OCTOBER, 1843.

---

### PART FIRST.—Analytical and Critical Reviews.

PAGE

ART. I.—1. De la Peste Orientale, d'après les matériaux recueillis à Alexandrie, au Caire, à Smyrne, et à Constantinople, pendant les années 1833-38. Par A. F. BULARD, de Méru; chargé de mission par le gouvernement français pour l'observation de la Peste dans toutes les localités de l'Empire Ottoman; inspecteur du service de la marine Egyptienne; et médecin en chef de l'hôpital militaire du Caire, etc. etc. . . . . . 289

On the Oriental Plague, from materials collected in Alexandria, Cairo, Smyrna, and Constantinople, during the years 1833-38. By A. F. BULARD, of Méru; Commissioner appointed by the French government for the observation of the Plague throughout the Ottoman Empire; Inspector of the Egyptian Navy; and chief Physician of the Military Hospital of Cairo, &c. &c.

2. De la Peste observée en Egypte. Recherches et Considérations sur cette Maladie. Par CLOT-BEY . . . . . . ib.

Researches and Considerations on the Plague as observed in Egypt. By CLOT-BEY.

3. Notes and Observations on the Ionian Islands and Malta, with some Remarks on Constantinople and Turkey, and on the System of Quarantine as at present conducted. By JOHN DAVY, M.D. F.R.SS.L. and E. Inspector-general of Army Hospitals . . . . . . ib.

4. Rapport addressé à S. E. le Ministre de l'Agriculture et du Commerce, sur des Modifications à apporter aux Réglements sanitaires. Par M. DE SÉGUR-DUPEYRON, Secrétaire du Conseil supérieur de Santé, etc. . . ib.

Report addressed to his Excellency the Minister of Agriculture and Commerce, on Modifications of the Sanatory Regulations. By M. DE SÉGUR-DUPEYRON, Secretary of the Superior Council of Health, &c.

5. Elements of Medicine. Vol. II.—Morbid Poisons. By ROBERT WILLIAMS, M.D., President of the Royal Medical and Chirurgical Society, and Senior-Physician of St. Thomas's Hospital, &c. . . . . ib.

6. Rapporto officiale fatto al sopra intendente alla Quarantina di alcuni casi di Peste. Scritto da LUIGI GRAVAGNA, M.D., medico principale di Sanita . ib.

Official Report of certain cases of Plague made to the Superintendent of Quarantine. By L. GRAVAGNA, M.D., Principal Physician of the Quarantine Establishment.

7. Lettre première au sujet des Accidents de Peste survenus tant au Lazaret de Koulély qu'a l'île de Proti, &c. Par ANTOINE PEZZONI, M.D., Membre du Conseil supérieur de Santé Ottoman, etc. . . . . ib.

First Letter on Cases of Plague which occurred in the Lazaretto of Koulély and the Isle of Proti, &c. By A. PEZZONI, M.D., Member of the Turkish Superior Council of Health, &c.

8. The Quarantine Laws, their abuses and inconsistencies; a Letter addressed to Sir John C. Hobhouse, M.P., President of the Board of Control. By A. T. HOLROYD, Esq. . . . . . . ib.

9. Report from the Select Committee of the House of Commons on the Contagion of Plague . . . . . . . . ib.

§

PAGE

ART. II.—Guy's Hospital Reports. Edited by G. H. BARLOW, M.D., and J. P. BABINGTON, M.A. Vol. VII. (Nos. XIV-XV.) . . . 308

1. Dr. BARLOW on the diagnosis of disease of the kidney . . ib.

2. Mr. TAYLOR'S report of a case of infanticide . . . 309

3. JOHN C. W. LEVER, Esq. on pelvic tumours obstructing parturition . 310

4. Mr. T. WILKINSON KING on the digestive solution of the œsophagus . ib.

5. Mr. EDWARD COCK on injury of the head relieved by operation . 312

6. Dr. GOLDING BIRD on urinary concretions and deposits . . ib.

7. Dr. H. MARSHALL HUGHES on urinary concretions and deposits . 317

8. Mr. C. ASTON KEY on a case of injured intestine . . . 319

9. Mr. FRANCE on a case of irideremia, or absence of the iris . . . 320

10. Dr. BABINGTON on a case of distended gall-bladder . . ib.

11. Dr. H. M. HUGHES on pneumonia . . . . ib.

12. Dr. C. W. LEVER on hemorrhage after delivery . . . ib.

13. Dr. GOLDING BIRD on the microscopic globules found in urine . 321

14. Mr. HILTON'S case of poisoning by arsenic . . . 322

15. Mr. W. G. CARPENTER'S case of fatal pleuritis . . . ib.

16. Mr. S. R. BEDFORD on inflammation of the eye . . . ib.

17. Dr. NORMAN CHEVERS on diseases of the aorta . . . 323

18. Mr. W. MURIEL on a case of contracted aorta . . . 326

19. Mr. HILTON on disease of the larynx . . . . ib.

20. Mr. MORGAN on the operation for cataract . . . ib.

21. Dr. G. H. BARLOW on diseases in early youth . . . ib.

ART. III.—Semeiotique des Urines, ou Traité des Altérations de l'Urine dans les Maladies; suivi d'un Traité de la Maladie de Bright aux divers ages de la vie. Par ALFRED BECQUEREL, M.D. &c. . . . . 328

Urinary Semeiology, or Treatise on the Changes of the Urine in Disease, &c. By ALFRED BECQUEREL, M.D.

ART. IV.—Deformities of the Spine and Chest, successfully treated by Exercise alone, and without Extension, Pressure, or Division of Muscles. By CHAS. H. ROGERS-HARRISSON, M.R.C.S. &c. &c. . . . . 341

ART. V.—Dr. Tavernier's Treatise on the Treatment of Deformities of the Spine by the "Lever-belt with Inclination Busk," without Extension Beds or Crutches; containing a Relation of new results obtained, and the Class of Cases in which the Belt may be safely applied. Translated into English, with a Critical Analysis of this Division of Orthopædia, by W. BREWER, M.D. 347

ART. VI.—The Bengal Dispensatory and Companion to the Pharmacopœia, chiefly compiled from the works of Roxburgh, Wallich, Ainslie, Wight and Arnot, Royle, Pereira, Lindley, Richard, Feé, and including the results of numerous special experiments. By W. B. O'SHAUGHNESSY, M.D., Assistant Surgeon to the Bengal Army, &c. &c., Professor of Chemistry and Materia Medica in the Medical College of Calcutta. Published by order of the Bengal Government . . . . . . . 352

2. Elements of Materia Medica and Therapeutics, including the recent discoveries and analysis of Medicines. By ANTHONY TODD THOMSON, M.D. F.L.S. &c. ib.

3. The Elements of Materia Medica and Therapeutics. By JONATHAN PEREIRA, M.D. F.R.S. & L.S. &c. . . . . . . ib.

ART. VII.—Recherches expérimentales sur les Propriétés et les Fonctions du Système Nerveux dans les Animaux Vertébrés. Par P. FLOURENS, Membre de l'Académie Française, Secrétaire perpétuel de l'Académie Royale des Sciences, etc. etc. . . . . . . . 364

Experimental Researches on the Properties and Functions of the Nervous System in Vertebrated Animals. By P. FLOURENS, Member of the French Academy, Perpetual Secretary of the Royal Academy of Sciences, &c. &c.

ART. VIII.—Recherches d'Anatomie comparée sur le Chimpansé. Par W. VROLIK, &c. &c. . . . . . . 373

Researches into the Comparative Anatomy of the Chimpansé. By W. VROLIK, &c. &c.

PAGE

ART. IX.—1. Beobachtungen auf dem Gebiete der Pathologie und Pathologischen Anatomie, gesammelt von Dr. JOH. FRIED. HERM. ALBERS, Professor der Medezin, &c. in Bonn . . . . . . 381
Observations on the Department of Pathology and Pathological Anatomy, collected by Dr. J. F. H. ALBERS, Professor of Medicine, &c. at Bonn.

2. Waarnemingen in het Gebied der Pathologie an der Pathologische-Anatomie. Door Dr. J. F. KERST, Chir. Mazoor, &c. . . . . ib.
Observations in the Department of Pathology and of Pathological Anatomy. By Dr. J. F. KERST, Surgeon-major, &c.

3. Bijdrage tot de Ontleedkundige Ziektekennis. Van F. S. ALEXANDER, Med. Doct. en Prof. to Utrecht . . . . . ib.
Contributions to Anatomical Pathology. By F. S. ALEXANDER, M.D., and Professor of Medicine at Utrecht.

ART. X.—Cases of Peritoneal Section for the Extirpation of Diseased Ovaria, by the large incision from Sternum to Pubes, successfully treated. By CHARLES CLAY, Member of the Royal College of Physicians, &c. . . 387

ART. XI.—J. B. van Helmont's System der Medicin, verglichen mit den bedeutenderen Systemen älterer und neuerer Zeit: ein Beitrag zur Entwickelungsgeschichte medicinischen Theorien; nebst der Skizze einer Theorie der Lebensercheinungen. Von Dr. G. A. SPIESS . . . 403
J. B. van Helmont's System of Medicine compared with the more remarkable Systems of ancient and modern Time; a contribution to the history of the development of medical theories; together with a sketch of a theory of the phenomena of life. By Dr. G. A. SPIESS, Practising Physician.

ART. XII.—On Spasm, Languor, Palsy, and other Disorders, termed Nervous, of the Muscular System. By JAMES ARTHUR WILSON, M.D., Fellow of the College of Physicians, and Physician to St. George's Hospital . . 418

ART. XIII.—Recherches Anatomiques, Pathologiques et Thérapeutiques sur la Phthisie. Par P. C. A. LOUIS, Médecin de l'Hôtel-Dieu, &c. . . 425
Anatomical, Pathological, and Therapeutical Researches on Phthisis. By P. C. A. LOUIS, Physician to the Hôtel-Dieu, &c. &c.

ART. XIV.—Practical Remarks on Gout, Rheumatic Fever, and Chronic Rheumatism of the Joints; being the substance of the Croonian Lectures for the present year, delivered at the College of Physicians. By ROBERT BENTLEY TODD, M.D. F.R.S., Physician to the King's College Hospital, and Professor of Physiology in King's College, London . . . . 460

ART. XV.—1. Allgemeine Krankheitslehre. Von Dr. K. F. H. MARX, ordentlichem Professor der Medicin in Göttingen, &c. . . . 472
General Pathology. By Dr. K. F. H. MARX, Ordinary Professor of Medicine at Göttingen.

2. Grundzüge zur Lehre von der Krankheit und Heilung. Von Dr. K. F. H. MARX, &c. . . . . . . . ib.
Elements of Pathology and Therapeutics. By Dr. K. F. H. MARX, &c.

ART. XVI.—On the Nature and Treatment of Stomach and Renal Diseases; being an Inquiry into the Connexion of Diabetes, Calculus, and other Affections of the Kidney and Bladder, with Indigestion. By WILLIAM PROUT, M.D. F.R.S. &c. . . . . . . . . 477

ART. XVII.—Ueber spontane und congenitale Luxationen, sowie über einen neuen Schenkelhalsbruch-Apparat. Von Dr. J. HEINE . . . 486
A Treatise on Spontaneous and Congenital Dislocations of the Hip, with a description of a new Apparatus for Fracture of the Neck of the Thigh-bone. By Dr. J. HEINE.

ART. XVIII.—Pulmonary Consumption, successfully treated with Naphtha. By JOHN HASTINGS, M.D., Senior Physician to the Blenheim-Street Free Dispensary . . . . . . . . 490

ART. XIX.—Brighton, and its three Climates; Remarks on its Medical Topography, and Advice and Warnings to Invalids and Visitors. By A. L. WIGAN, M.D. Surgeon, formerly practising in that Town . . . 500

PAGE

Art. XX.—Physiologie der Entzündung und Regeneration in den organischen Geweben. Von Dr. Hermann Klencke . . . . 503

Physiology of Inflammation and Regeneration in the Organic Tissues. By Dr. Hermann Klencke.

### Part Second.—Bibliographical Notices.

Art. I.—Some Account of the African Remittent Fever which occurred on board Her Majesty's Steam-ship Wilberforce, in the River Niger, and whilst engaged on Service on the Western Coast of Africa; comprising an Inquiry into the Causes of Disease in Tropical Climates. By Morris Pritchett, M.D. F.R.G.S., Member of the Royal College of Physicians of London, Fellow of the Royal Medical and Chirurgical Society, late Surgeon of H.M. Ship Wilberforce, &c. . . . . . . . 505

Art. II.—Thèse sur le Delirium Tremens, ou Folie des Ivrognes, &c. Par le Docteur Bougard . . . . . . 506

Thesis on Delirium Tremens, or the Madness of Drunkards, &c. By Dr. Bougard.

Art. III.—Ueber das Verhaltniss der Medicin zur Chirurgie, und die Dreiheit in heilenden Staate, &c. Von Dr. C. H. Bischoff, &c. . . 507
On the Relation of Medicine to Surgery, and on the threefold Division of the Medical Profession. By Dr. C. H. Bischoff, &c.

Art. IV.—Facts and Observations relative to the Influence of Manufactures upon Health and Life. By Daniel Noble, Surgeon . . . ib.

Art. V.—Darstellung der Aequilibrial-Methode zur sicheren Heilung der oberschenkelbrüche ohne Verkurzung. Von Georg Mojsisovics, Doctor der Medicin und Chirurgie, Primarchirurgen im k. k. allgemeinen Krankenhause . 508
An account of the Æquilibrial Treatment employed for the more successful union of Fractures of the Thigh-Bone, and the Prevention of any Shortening of the Limb. By George Mojsisovics, Doctor of Medicine and Surgery, Senior Surgeon of the Imperial Hospital at Vienna, &c.

Art. VI.—A Diagram to define the Lives of the Patriarchs, and the early History of the Seed of the Serpent and the Seed of the Woman, particularly in reference to the origin of disease and the danger of unsanctified knowledge. By H. L. Smith, M.R.C.S., &c. . . . . . 510

Art. VII.—Tic Douloureux, or Neuralgia Facialis, and other Nervous Affections: their Seat, Nature, and Cause. With Cases illustrating successful methods of Treatment. By R. H. Allnatt, M.D. A.M. F.S.A. Second Edition . 511

Art. VIII.—Mental Hygiene, or an examination of the Intellect and Passions, designed to illustrate their influence on Health and the duration of Life. By William Sweetser, M.D. . . . . . . ib.

Art. IX.—A Practical Treatise on the Diseases peculiar to Women. By Samuel Ashwell, M.D. Part II. Organic Diseases . . . . 512

Art. X.—Mémoire sur l'Emploi du Carbonate d'Ammoniaque dans la Scarlatine. Par le Dr. Rieken . . . . . . ib.

Memoire on the Employment of Carbonate of Ammonia in Scarlatina. By Dr. Rieken.

### Part Third.—Original Reports and Memoirs.

1. On the Ovum of Man and the Mammifera before and after Fecundation. By T. Wharton Jones, F.R.S. &c. &c. . . . . . 513

2. On the Epidemic Ague or "Fainting Fever" of Persia, a Species of Cholera, occurring in Teheran in the Autumn of the year 1842. By Charles W. Bell, M.D., Physician to Her Majesty's Mission to Persia . . 558

Books received for Review . . . . . 566

Title, Index, &c.

